# Development of a Semi-Automatic Segmentation Method for Retinal OCT Images Tested in Patients with Diabetic Macular Edema

**DOI:** 10.1371/journal.pone.0082922

**Published:** 2013-12-26

**Authors:** Yijun Huang, Ronald P. Danis, Jeong W. Pak, Shiyu Luo, James White, Xian Zhang, Ashwini Narkar, Amitha Domalpally

**Affiliations:** 1 Fundus Photograph Reading Center, Department of Ophthalmology and Visual Sciences, University of Wisconsin-Madison, Madison, Wisconsin, United States of America; 2 McPherson Eye Research Institute, University of Wisconsin-Madison, Madison, Wisconsin, United States of America; 3 Department of Psychiatry, Yale University, New Haven, Connecticut, United States of America; University of Düsseldorf, Germany

## Abstract

**Purpose:**

To develop EdgeSelect, a semi-automatic method for the segmentation of retinal layers in spectral domain optical coherence tomography images, and to compare the segmentation results with a manual method.

**Methods:**

SD-OCT (Heidelberg Spectralis) scans of 28 eyes (24 patients with diabetic macular edema and 4 normal subjects) were imported into a customized MATLAB application, and were manually segmented by three graders at the layers corresponding to the inner limiting membrane (ILM), the inner segment/ellipsoid interface (ISe), the retinal/retinal pigment epithelium interface (RPE), and the Bruch's membrane (BM). The scans were then segmented independently by the same graders using EdgeSelect, a semi-automated method allowing the graders to guide/correct the layer segmentation interactively. The inter-grader reproducibility and agreement in locating the layer positions between the manual and EdgeSelect methods were assessed and compared using the Wilcoxon signed rank test.

**Results:**

The inter-grader reproducibility using the EdgeSelect method for retinal layers varied from 0.15 to 1.21 µm, smaller than those using the manual method (3.36–6.43 µm). The Wilcoxon test indicated the EdgeSelect method had significantly better reproducibility than the manual method. The agreement between the manual and EdgeSelect methods in locating retinal layers ranged from 0.08 to 1.32 µm. There were small differences between the two methods in locating the ILM (p = 0.012) and BM layers (p<0.001), but these were statistically indistinguishable in locating the ISe (p = 0.896) and RPE layers (p = 0.771).

**Conclusions:**

The EdgeSelect method resulted in better reproducibility and good agreement with a manual method in a set of eyes of normal subjects and with retinal disease, suggesting that this approach is feasible for OCT image analysis in clinical trials.

## Introduction

Optical Coherence Tomography (OCT) - determined retinal layer thickness measurements have used in clinical trials as quantitative, morphologic endpoints for the diagnosis and classification of retinal diseases and for monitoring treatment effects [1], [2], [3], [4], [5], [6], including studies in patients with diabetic macular edema. In order for the retinal layer thickness be measured properly, accuracy of the applied layer segmentation method becomes an important determinant especially when structural-altering retinal lesion is present. With the advent of spectral domain OCT, a number of research groups and manufacturers have engaged in developing layer segmentation strategies [7], [8], [9], [10], [11], [12], [13]. To date, published and commercially available segmentation methods fall into two groups, either fully automated or manual. With a fully-automated method, computer algorithm determines the desired layers with no human supervision. While it is very convenient and practical in the traditional clinical practice settings, the segmentation results using automatic algorithms are prone to layer misidentification errors, especially in eyes with intermediate to severe retinal lesions where the error rate ranges from 30% to 80% [14], [15], [16], [17], [18]. The layer misidentification, while confounded by other OCT imaging and operating errors such as weak signal quality, eye movement, and decentration, are mostly caused by the complexity of morphological configuration and reflectivity changes of retinal lesions that are beyond the reach of traditional image segmentation techniques.

Manual segmentation by human graders has been considered the “gold standard” in many previous reports [12], [13], [19]. Manual segmentation by human graders usually requires the grader to identify the layers either by free-hand drawing [12], or placing seed points and the computer interpolating the layers via point-fitting algorithms [13]. While the gross errors of layer mis-identification frequented in the automated methods are avoided, the manual methods are usually time- and labor-intensive, and generally yield higher inter-grader variability.

Taking into consideration the advantages and drawbacks of both approaches, we developed a semi-automatic, interactive segmentation method (called herein EdgeSelect). We tested the performance of the EdgeSelect method and compared it against a manual method for detection of the inner and outer boundaries of the retina.

## Methods

### Study dataset and preparation

**Figure 1:**
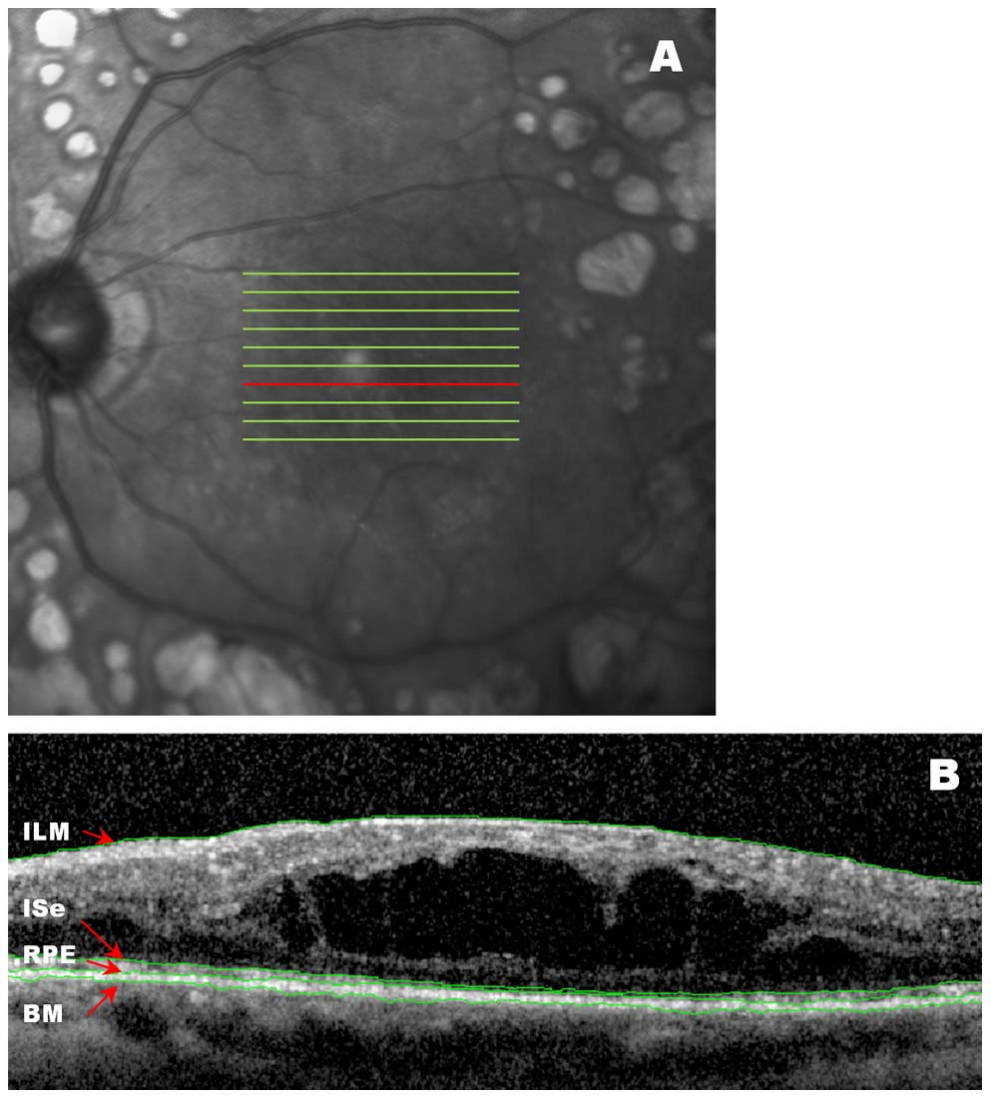
A representative OCT image with layers segmented using EdgeSelect. (**A**) OCT images were captured with horizontal raster lines covering 4.5×6 mm area centered at the fovea. (**B**) A representative B scan is shown by the red line in (A). The inner limiting membrane (ILM), the inner-segment/ellipsoid interface (ISe), the retinal/retinal pigment epithelium interface (RPE), and the Bruch's membrane (BM), were segmented.

Retina volume scans from 28 eyes consisting of 4 normal subjects and 24 patients with diabetic macular edema were obtained using Heidelberg Spectralis OCT devices (Heidelberg Engineering Inc, Heidelberg, Germany). The patient scans were provided by the Diabetic Retinopathy Clinical Research Network (DRCR.net) from participants enrolled in a study comparing the measurements from spectral domain and time domain OCTs. The volume scans consist of horizontal raster lines covering a 4.5×6 mm area centered at the fovea (Figure 1A), of which each B scan was captured with 9-frame averaging (ART = 9). Scans were obtained with certified photographers to minimize the OCT data acquisition artifacts [15], [20]. The data samples were saved in the Heidelberg proprietary .e2e format. They were exported from a Heidelberg Heyex review software (version 5.1) in .vol format and converted to the DICOM (Digital Imaging and Communication in Medicine) [21] OPT (ophthalmic tomography) format using a custom application built in MATLAB (MATLAB R2011b, The MathWorks, Natick, MA). The standardized OCT images were then segmented by three graders independently for the layers of the inner limiting membrane (ILM), the inner segment/ellipsoid interface (ISe), the retina/retinal pigment epithelium interface (RPE), and Bruch's membrane (BM), using both manual and EdgeSelect segmentation methods (Figure 1B).

The study was conducted in accordance with Health Insurance Portability and Accountability Act (HIPAA) requirements and the tenets of the Declaration of Helsinki. All images were obtained under informed written consent and de-identified through the DRCR.net clinical trial protocol, and the study protocol was approved by the institutional review board of the University of Wisconsin-Madison.

### Segmentation Procedures

#### The manual method

The manual segmentation method used in this study was similar to the method described by Hood, et. al. [13] Graders used the computer mouse to place the seed points at a desired OCT layer, and the computer automatically interpolated the layer based on a spline fitting algorithm. The graders continued to add/delete seed points until the resultant spline line adequately demarcated the layer. The graders segmented each of the four layers for all B-scans in the volumetric data before continuing on the next layer.

#### The EdgeSelect method

Several weeks after the graders performed the manual segmentation of the scans, they re-segmented them for the same four layers using the EdgeSelect method.

**Figure 2:**
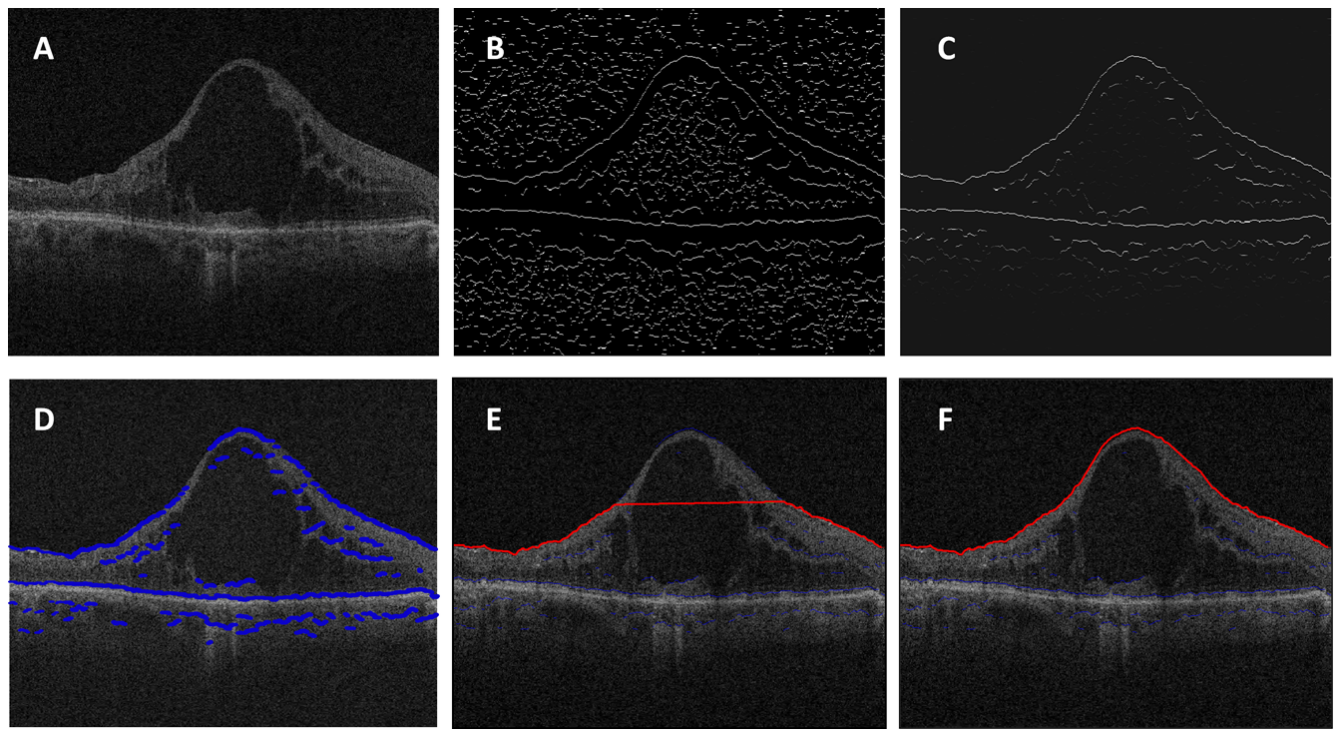
Graphic representation of segmenting the inner limiting membrane (ILM) interface in a representative B scan using the EdgeSelect method. (**A**) A representative OCT B scan. (**B**) Detection of image contrast change using zero-crossing of a Laplacian-of-Gaussian (LoG) filter. (**C**) The detected edges are assigned different weights based on intensity/gradient characteristics. Higher intensity represents larger weight, corresponding to strong edges; lower intensity signals are assigned lesser weight corresponding to weak edges. (**D**) An edge candidate map (blue lines) is generated using a Canny-like filtering scheme, and is superimposed on the original OCT image. (**E**) The start and end edge candidates are initiated, and the path of the shortest distance via Dijkstra's algorithm (red line) is computed. (**F**) Human grader intervention adds additional seed edges, and the program regenerates automatically the updated shortest path until the proper layer segmentation is reached.

The general steps of the EdgeSelect method to generate the retinal layers are illustrated and described in Figure 2 by segmenting the ILM layer in a representative B-scan. It is of note that the steps leading to interactive edge selection and layer generation are based on automatic image processing routines and require no grader intervention.

1. To reduce speckle noise, OCT images were smoothed by applying a 3×3 pixel boxcar averaging filter.
2. The layer locations in OCT images were defined as the transition between two regions with difference in reflectance intensity, which were identified in EdgeSelect as the zero-crossing pixels when a Laplacian of Gaussian filter was applied [22]. The zero-crossing pixels correspond to the local maximum gradient in intensity. Figure 2B shows the zero-crossing binary image derived from the original OCT image.
3. Each of the zero-crossing pixels were assigned values with a weighting scheme based on the intensity and gradient of the neighboring pixels. The weighting scheme was designed based on the characteristics of the desired layer. For ILM, a gradient filter enhancing the transition from low reflectivity to high reflectivity was used. The weighted zero-crossing map was plotted in Figure 2C; many spurious edges were suppressed by the weighting scheme.
4. The weighted zero-crossing map was then applied with a Canny-edge detection scheme [23]: only the pixels connected with pixels with strong weight were considered as edge. Figure 2D illustrates the edge candidates superimposed on the original OCT B-scan.
5. To generate the layer location, EdgeSelect used the Dijkstra's algorithm [24] interactively based on the edges selected by the user. The program automatically selected one edge candidate from the leftmost of the image as the “source”, and one from the rightmost of the image as the “destination”, and generated the shortest path from the “source” to the “destination”. The choices of the “source” and “destination” edges depends on the relative position of the desired layer within the retinal tissue. For example, if ILM layer is segmented, the program will choose the edges near the vitreal high reflectivity band; if ISe, RPE, or BM layers are segmented, the program will choose the edges near the posterior high reflectivity band. If the generated layer was satisfactory to demarcate the desired layer, no further human intervention was necessary. However, in diseased retinas where the layered structure was altered, the grader could pick additional or remove edge candidates from the initial set for the Dijkstra's algorithm to re-route the shortest path until the resultant layer was correct. This process is illustrated in Figure 2E and 2F. Figure 2E shows the resultant ILM layer from initial automatic edge selection. Because of the presence of a large cyst in the OCT image, the initial shortest-path failed to demarcate correctly the layer on the center portion of the image. The grader intervened by selecting the correct edge on the top of the cyst, and the ILM layer was recalculated automatically with the inclusion of the new edge candidate (Figure 2F).

### Data Analysis

Comparison of the manual and EdgeSelect methods was performed by evaluating the inter-grader reproducibility for each method, and the agreement of the layer locations between the two methods. The inter-grader reproducibility of the mean layer location was calculated with concordance correlation coefficients. In addition, we also evaluated the variability at the pixel level. For a particular layer, the absolute difference in boundary location (ΔBL) at each pixel was calculated between any grader pairs, and the inter-grader reproducibility was defined as the value of the ΔBL averaged across the entire 3D volumetric data and among the three grader pairs. Scatter plots of the inter-grader reproducibility of each data sample was used to compare the manual and EdgeSelect method visually, and Wilcoxon signed rank test [25] was used to determine if the inter-grader reproducibility between the two methods differed, assuming the data sample distribution was not normal.

We were also interested in determining whether the layers were accurately segmented using the EdgeSelect method, i.e., if the layer locations identified by the EdgeSelect method were in agreement with those by the manual method. At each pixel, we computed the layer location averaged among the three graders for the EdgeSelect and the manual methods independently. The agreement was measured by computing the absolute difference of the mean layer locations between the manual and the EdgeSelect methods at each pixel, and then averaged across the 3D data. Similarly, scattered plot of agreement of each data sample was used for visual inspection, and Wilcoxon signed rank test was used to determine the statistical difference between the two measurements.

## Results

### Inter-grader reproducibility

**Figure 3:**
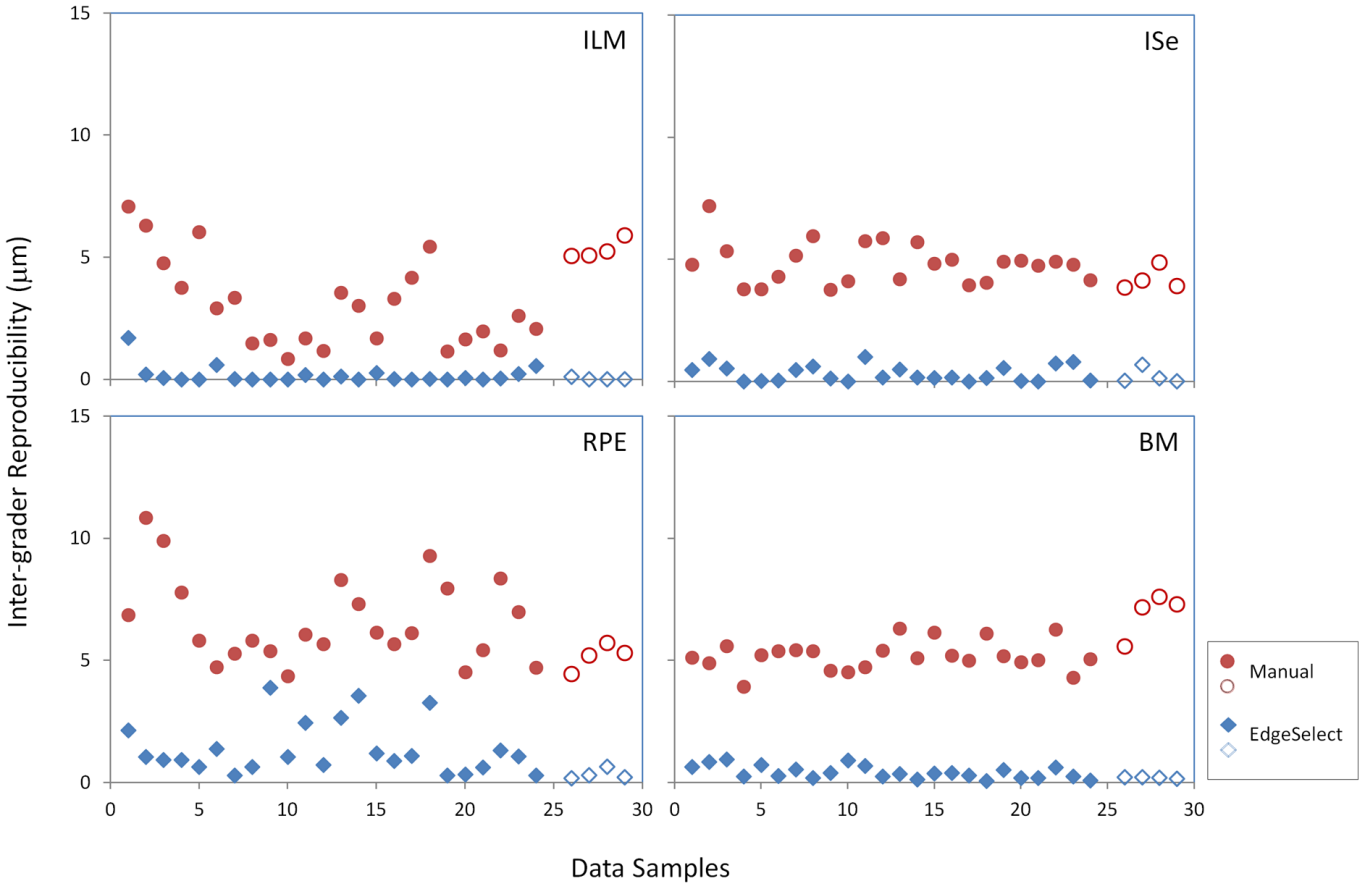
Comparison of the inter-grader reproducibility between the manual and the EdgeSelect methods in all 28 data samples. The filled symbols are data points from patients and the open symbols are from normal subjects. ILM: inner limiting membrane; ISe: the inner-segment/ellipsoid interface; RPE: the retinal/retinal pigment epithelium interface; BM: the Bruch's membrane.

Both the Manual and EdgeSelect methods exhibited high concordance correlation among graders in identifying the mean layer locations of the ILM, ISe, RPE and BM (Table 1). At the pixel level, reproducibility for the EdgeSelect method varied from 0.15 to 1.22 µm; in contrast, reproducibility for the manual method ranged from 3.36 to 6.43 µm. To further illustrate the difference in reproducibility between these two methods, the scatter plots of reproducibility for the four layers are shown in Figure 3. In each of the 28 data samples, reproducibility of the EdgeSelect method pixel-wise was better than that of the manual method. Wilcoxon test indicated the improvement was statistically significant (p<0.001) for each layer.

**Table 1:** Mean, standard deviation, and concordance correlation of the inter-grader reproducibility of the EdgeSelect and the Manual methods.

	Layer	Mean (µm)	Std. Dev. (µm)	Concordance Corr.
EdgeSelect	ILM	0.15	0.34	.99
	ISe	0.31	0.32	.99
	RPE	1.22	1.05	.99
	BM	0.39	0.26	.99
Manual	ILM	3.36	1.85	.99
	ISe	4.75	0.83	.99
	RPE	6.43	1.71	.99
	BM	5.45	0.88	.99

Layers are: inner limiting membrane (ILM), inner segment/ellipsoid interface (ISe), retina/retinal pigment epithelium interface (RPE), and Bruch's membrane (BM).

It was also noted that the inter-grader reproducibility reported here was similar in magnitude to the MEAN (mean(ΔLBL)) reported by Hood *et. al.* [13], in which it was reported to be 1.9–4.0 µm. Since the MEAN (mean(ΔLBL)) by Hood *et. al.* was defined as the absolute difference between grader and the mean location of all graders, and in our report the inter-grader reproducibility was defined as the absolute difference between grader pairs, the value of the inter-grader reproducibility is twice as large as the MEAN (mean(ΔLBL)). Not surprisingly, the range of the inter-grader reproducibility for the manual method, 3.36–6.43 µm, was congruent with those reported by Hood et. al., as the manual methods employed in these two reports were virtually identical.

### Agreement

**Figure 4:**
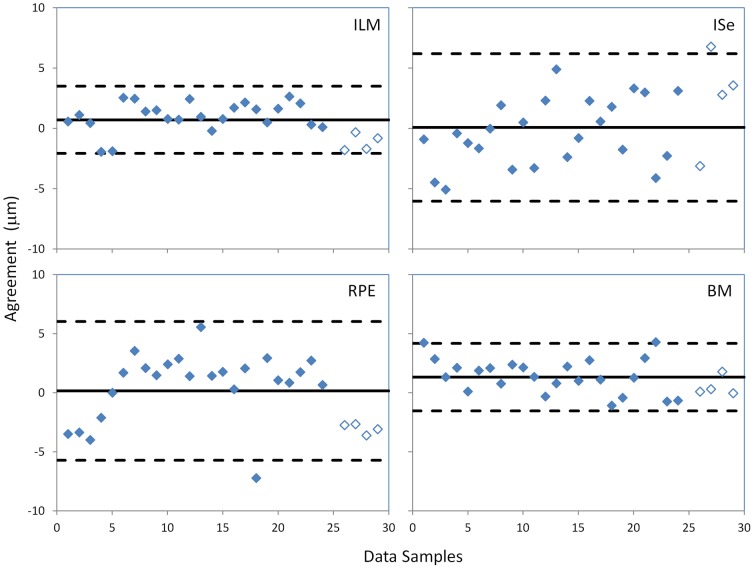
Agreement in segmented layer locations between the manual and EdgeSelect methods. Solid lines indicate the mean difference of the agreement between the two methods and the dashed lines indicate mean ± 2 standard deviations. The filled symbols are data points from patients and the open symbols are from normal subjects. ILM: inner limiting membrane; ISe: the inner-segment/ellipsoid interface; RPE: the retinal/retinal pigment epithelium interface; BM: the Bruch's membrane.

Figure 4 shows the agreement in identifying layer locations between the manual and EdgeSelect methods. The mean difference for the ILM, ISe, RPE, and BM layers were 0.71±1.39 µm (mean ± 1 std. dev.), 0.08±3.06 µm, 0.16±2.94 µm, and 1.32±1.43 µm, respectively. There was a small difference between the manual and EdgeSelect methods in identifying the ILM (p = 0.012, Wilcoxon test) and BM layers (p<0.001), but the ISe and RPE layers were statistically indistinguishable between the two methods (p = 0.896 and p = 0.771). The small difference between the two methods in locating the ILM and BM layers was presumably due to the disparity that EdgeSelect method objectively found the location of the local maximum gradient in reflectance, while the results of the manual method were influenced by the human perception of an edge, in particular where there was an asymmetrical intensity profile of the neighboring pixels.

Additional analysis is done for comparing the inter-grader variability and agreement in patient and normal subject groups independently. The results is provided as Table S1.

## Discussion

Accurate segmentation of OCT retinal images has been a challenge for OCT device manufacturers and research groups. An ideal algorithm or strategy incorporates elements to ensure high accuracy in layer identification in various clinical conditions as well as low inter-session variability. Among the existing methods, automatic segmentation algorithms have the computational superiority and usually produce perfect inter-session reliability when applied to the same OCT image. However, in diseased eyes where retinal lesions are heterogeneous and complex, automated methods frequently fail to identify retinal layers properly, and the human observer remains the best decision-maker for definition and control of the desired segmented results. In contrast, manual methods usually have limited layer misidentification errors, but tend to have high inter-grader variability and are usually time and effort consuming.

In this report, we described the EdgeSelect method, which allows graders to incorporate their clinical knowledge to guide the selection of the proper edge candidates of a particular layer, but delegates the calculation of the exact pixel location of the layer path to the computer via using the Dijkstra's algorithm interactively. When comparing with a manual method, the EdgeSelect method demonstrated better inter-grader reproducibility, while maintaining good agreement with the manual method. When compared to an automatic method, the EdgeSelect method should perform at the same level of efficiency in the retinas with normal or near-normal structure, as the initial automatic edge selection is likely accurate and hence no human intervention is needed. In diseased retinas, the advantage of the interactive nature of the EdgeSelect method becomes evident, especially where patchy or locally discontinuous layer signals are common. Using EdgeSelect, we anticipate increased efficiency in layer segmentation, which should allow segmentation of large 3D data sets to become feasible for graders.

Lastly, the EdgeSelect method relies on local transition of the reflectance signal to determine the proper edges, which is less likely to be device dependent. Together with methods of data standardization and homogenization [26], EdgeSelect can be applied universally to images from different SD-OCT devices, which would allow all OCT images to be processed using the same software algorithm for harmonization of the measurements.

## Supporting Information

Table S1 The inter-grader variability and agreement between EdgeSelect and manual measurement is analyzed independently for the patient and normal subjects groups.
(DOCX)
