# Supplementary material for: Development of a Semi-Automatic Segmentation Method for Retinal OCT Images Tested in Patients with Diabetic Macular Edema
Source: PLoS One. 2013 Dec 26;8(12):e82922. doi: 10.1371/journal.pone.0082922 (PMC3873283; doi:10.1371/journal.pone.0082922)
Supplement: Table S1 — The inter-grader variability and agreement between EdgeSelect and manual measurement is analyzed independently for the patient and normal subjects groups. (DOCX) [file pone.0082922.s001.docx]

**Table S1:**

|  |  | Patients | | Normal Subjects | |
| --- | --- | --- | --- | --- | --- |
|  | (μm) | EdgeSelect | Manual | EdgeSelect | Manual |
| Inter-grader | ILM | 0.170 | 3.033 | 0.027 | 5.316 |
| variability | ISe | 0.327 | 4.838 | 0.212 | 4.196 |
|  | RPE | 1.366 | 6.636 | 0.336 | 5.163 |
|  | BM | 0.426 | 5.202 | 0.195 | 6.918 |
| Agreement | ILM | 1.023 | | -1.156 | |
|  | ISe | -0.328 | | 2.500 | |
|  | RPE | 0.693 | | -3.020 | |
|  | BM | 1.450 | | 0.535 | |

The inter-grader variability and agreement between EdgeSelect and manual measurement is analyzed independently for the patient and normal subjects groups.
